# Outcomes of allogeneic hematopoietic stem cell transplantation in R/R DLBCL patients with failure of CAR-T therapy

**DOI:** 10.1186/s40164-024-00473-y

**Published:** 2024-01-16

**Authors:** Mengya Cong, Sicheng Ai, Liqing Kang, Mao Jin, Ying Zhu, Caixia Li, Zhengming Jin, Lei Yu, Depei Wu, Haiwen Huang

**Affiliations:** 1 National Clinical Research Center for Hematologic Diseases, The First Affiliated Hospital of Soochow University, Suzhou, China; 2 College of Art and Science, New York University, New York City, USA; 3 Shanghai Unicar-Therapy Biomed-Phamaceutical Technology CO, LTD, Shanghai, China

**Keywords:** R/R DLBCL, CAR-T, Allogeneic hematopoietic stem cell transplantation, Retrospective analysis

## Abstract

From October 2017 to June 2022, we retrospectively report outcomes of R/R DLBCL patients with failure of CAR-T therapy, then receiving allo-HSCT. Among 10 patients, 5 were males and 5 females, with a median age of 43.5 (27–52) years. All patients were diagnosed refractory/relapsed diffuse large B cell lymphoma. The median time from CAR-T treatment to transplantation was 84.5 (31–370) days. The median follow-up was 21 (3–69) months. 5/10 patients attained CR and 1/10 patient attained PR during the follow up. The objective response rate (ORR) was 60%. The 1-year overall survival (OS) and progression-free survival (PFS) were 70% and 40%, respectively. At the time of the analysis, 6 patients were still living. During the follow up, four patients have died and the causes were disease relapses and progressions (2 patients), acute renal failure (1 patient), severe pulmonary infection (1 patient). Non-relapse was 20.0%.

## To the editor

Refractory/relapsed diffuse large B-cell lymphoma (R/R DLBCL) patients with failure of CAR-T therapy have poor prognosis and no standard chemotherapy regimen has been defined [1]. Therefore, it is apparent that novel treatment modalities are urgently needed for them. For such patients, allogeneic hematopoietic stem cell transplantation (allo-HSCT) should be considered as a treatment option. An expert panel opinion from the American Society for Transplantation and Cellular Therapy suggests that allo-HSCT may be considered for patients with CR post CAR-T therapy after individualized evaluation, whereas in patients with relapse/progression allo-HSCT should be included among treatment options [2].

We performed a retrospective analysis of patients with R/R DLBCL who relapsed after CAR-T therapy at the First Affiliated Hospital of Soochow University. From October 2017 to June 2022, we report outcomes of R/R DLBCL patients with failure of CAR-T cell treatment, then receiving allo-HSCT. In our analysis, 10 patients’ clinical data were collected. All patients were diagnosed based on histopathologic examinations. Following CAR-T therapy, a response evaluation via positron emission tomography (PET)/computed tomography (CT) was performed. The response to treatment was evaluated according to the Lugano 2014 Evaluation criteria for therapeutic effect of lymphoma.

Among 10 patients, 5 were males and 5 females, with a median age of 43.5 (27–52) years. 8/10 patients were at stage IV and 2/10 were at stage III. IPI ranged from 2 to 4. IPI was 2 in four patients, 3 in five patients and 4 in one patient. All patients had received CAR-T therapy before allo-HSCT. Four patients received CD19-targeted CAR-T therapy, six patients received CD19/CD22-targeted CAR-T therapy. All of these CART cells were autologous CART. Best response to CAR-T therapy was CR in 30% (n = 3), PR in 40% (n = 4), and SD/PD in 30% (n = 3). Demographic and clinical characteristics are described in Table 1.

**Table 1:** Clinical features of patients (n = 10)

	Patient	
	No	%
Sex		
Male	5	50.0%
Female	5	50.0%
Age (years)		
Range	27–52	
Median	43.5	
ECOG performance stage		
0–1	7	70.0%
≥ 2	3	30.0%
Ann Arbor clinical stage		
III	2	20.0%
IV	8	80.0%
LDH higher than ULN	7	70.0%
Disease type		
DLBCL	10	100.0%
Prior therapies		
Range3–10		
Median	9	
IPI		
0–1	0	0
2	4	40.0%
3	5	50.0%
4	1	10.0%

During the follow up, all patients relapsed in several months after CAR-T therapy. The first-line treatment regimens given as salvage following CAR-T failure were: venetoclax-based (n = 2), ibrutinib+tislelizumab (n = 1). 7 patients received no therapy for their active lymphoma between CAR-T and allo-HSCT.

The median time from CAR-T therapy to transplantation was 84.5 (31–370) days. 8/10 donors were haploidentical family member, 2/10 donors were HLA-matched sibling. The conditioning regimens included BU/CY (busulfan, cyclophosphamide) and TBI/CY treatments. Bone marrow+peripheral blood was the most common graft source. The most common GVHD prophylaxis regimen was cyclosporinA+mycophenolate mofetil+methotrexate (90%). All patients obtained complete engraftment. Median time of neutrophil engraftment was 12 (11–19) days, and 13 (9–20) days of platelet engraftment. The CD34+ cells ranged from 0.95 × 106 to 7.36 × 10^6^cells/kg (median 3.04 × 10^6^ cells/kg). (Table 2).

**Table 2:** Treatment characteristics and response (n = 10)

Patient	Time (d)	Disease status prior to allo-HSCT	Type of donors	stem cell source	Conditioning regimens	CD34+ cell(× 10^6^cells/kg)	GvHD PPx	Efficacy
1	51	PR	MMRD	Peripheral blood	TBI/Cy	7.36	CsA+MMF+MTX	CR
2	370	PD	MMRD	Bone marrow+peripheral blood	Bu/Cy	0.95	CsA+MMF+MTX	PD
3	246	PD	MMRD	Bone marrow	Bu/Cy	3.87	CsA+MMF+MTX	CR
4	45	PD	MMRD	Bone marrow+peripheral blood	Bu/Cy	2.22	CsA+MMF+MTX	CR
5	101	PD	MMRD	Bone marrow+peripheral blood	Bu/Cy	2.64	CsA+MMF+MTX	PD
6	31	PD	MMRD	Bone marrow+peripheral blood	TBI/Cy	1.96	CsA+MMF+MTX	CR
7	50	PR	MMRD	Bone marrow+peripheral blood	Bu/Cy	5.49	CsA+MMF+MTX	PD
8	133	PR	MSD	Peripheral blood	Bu/Cy	3.68	CsA+MTX	PR
9	68	PD	MMRD	Bone marrow+peripheral blood	Bu/Cy	1.56	CsA+MMF+MTX	PD
10	119	PD	MSD	Peripheral blood	Bu/Cy	3.43	CsA+MMF+MTX	CR

Time The time from CAR-T treatment to transplantation, MMRD mismatched related donor, MSD matched sibling donor, Bu/Cy busulfan/cyclophosphamide, GvHD PPx graft-versus-host disease prophylaxis, CsA cyclosporinA, MMF mycophenolate mofetil, MTX methotrexate, CR complete remission, PR partial remission, PD progression disease.

The median follow-up was 21 (3–69) months. 5/10 patients attained CR and 1/10 patient attained PR during the follow up. The ORR was 60%. Median OS was 21 months (range 3–69 months), and 1-year OS was 70% for all patients. Median PFS was 5 months (range 0.7–62 months), and 1-year PFS was 40%.

1 case experienced acute graft-versus-host disease(aGVHD) grade II, 1 case with aGVHD grade III. Among 5 survivals, NIH mild chronic GVHD occurred in 1 patient. At time of last follow-up, four patients had died and the causes were disease relapses and progressions (2 patients), acute renal failure (1 patient), severe pulmonary infection (1 patient). Non-relapse was 20.0%.

CAR-T therapy have been proved as a most effective strategy for R/R DLBCL patents [3, 4]. However, there is no standard of care treatment options post CAR-T failure [5]. FDA-approved therapies for R/R LBCL which can be employed after CAR-T failure include polatuzumab vedotin plus bendamustine and rituximab, tafasitamab-lenalidomide and selinexor, which are not available in China [6]. Thus, to those who achieve a type of response post CAR-T failure, a consolidation with allo-HSCT may be considered. In the ZUMA-1 trial, 2 patients who had a response underwent allo-HSCT, while in the JULIET trial no patient proceeded to transplantation while having a response. However, 6 patients who were unresponsive to CAR T-cells proceeded ultimately to allo-HSCT [7, 8]. In a smaller study of 61 B-NHL patients with progressive disease after anti-CD19 CAR-T therapy, 5 patients (8%) eventually received an allo-HSCT with 2 of them remaining alive after 12 months of disease progression [9]. Zurko J et al. reported the outcome of 88 patients with relapsed, refractory LBCL received an allo-HSCT following anti-CD19 CAR-T failure. The 1-year OS, PFS, and graft-versus-host disease-free relapse-free survival were 59%, 45%, and 39% respectively [10]. Fried et al. reported the outcome of 39 adult LBCL patients who underwent allo-HSCT following anti-CD19 CAR-T therapy. The 2-year overall survival (OS) and progression-free survival (PFS) were 45% (95% CI 31% to 66%) and 31% (95% CI 19% to 50%), respectively [11]. We report 1-year OS and PFS of 70% and 40% following allo-HCT, respectively. This compares favorably with the dismal outcome of patients with CAR-T failure.

In conclusion, this study evaluated the efficacy and safety of allo-HSCT in a subset of DLBCL patients previously treated with CAR T-cell therapy, it has shown that allo-HSCT is a feasible and safe choice with favorable outcome for R/R DLBCL patients with failure of CAR-T therapy.

## Data Availability

The datasets used and analyzed during the current study are available from the corresponding author on reasonable request.

## Abbreviations

R/R DLBCL: Refractory/relapsed Diffuse large B-cell lymphoma
Allo-HSCT: Allogeneic hematopoietic stem cell transplantation
OS: Overall survival
PFS: Progression-free survival
CR: Complete remission
PR: Partial remission
PD: Progression disease
